# Metabolism of Tracer ^75^Se Selenium From Inorganic and Organic Selenocompounds Into Selenoproteins in Rats, and the Missing ^75^Se Metabolites

**DOI:** 10.3389/fnut.2021.699652

**Published:** 2021-07-12

**Authors:** Jacqueline K. Evenson, Roger A. Sunde

**Affiliations:** Department of Nutritional Sciences, University of Wisconsin, Madison, WI, United States

**Keywords:** glutathione peroxidase, SDS/PAGE, selenite, selenomethionine, selenosugar

## Abstract

We now know much about selenium (Se) incorporation into selenoproteins, and there is considerable interest in the optimum form of Se for supplementation and prevention of cancer. To study the flux of ^75^Se into selenoprotein, rats were fed 0 to 5 μg Se/g diet as selenite for 50–80 d and injected iv with 50 μCi of ^75^Se-labeled selenite, selenate, selenodiglutathione, selenomethionine, or selenobetaine at tracer levels (~0.5 μg Se). The rats were killed at various times and ^75^Se incorporation into selenoproteins was assessed by SDS/PAGE. These studies found that there is very rapid Se metabolism from this diverse set of selenocompounds to the common intermediate used for synthesis and incorporation of ^75^Se into the major selenoproteins in a variety of tissues. No selenocompound was uniquely or preferentially metabolized to provide Se for selenoprotein incorporation. Examination of the SDS/PAGE selenoprotein profiles, however, reveals that synthesis of selenoproteins is only part of the full Se metabolism story. The ^75^Se missing from the selenoprotein profiles, especially at early timepoints, is likely to be both low-MW and high-MW selenosugars and related precursors, as we recently found in livers of turkeys fed Se-adequate and high-Se diets. Differential metabolism of different selenocompounds into different selenosugar species may occur; these species may be involved in prevention of cancer or other diseases linked to Se status and may be associated with Se toxicity. Additional studies using HPLC-mass spectroscopy will likely be needed to fully flesh out the complete metabolism of selenium.

## Introduction

We now know much about selenium (Se) incorporation into selenoproteins. Se at the selenide level is metabolized to selenophosphate, esterified to serine while attached to a novel selenocysteine tRNA, and incorporated into the selenoprotein backbone during translation at the position specified by a UGA codon and requiring a 3'UTR stem-loop selenocysteine (Sec) insertion sequence (1–6). At the time the experiments here were conducted, only five mammalian selenoproteins had been identified and characterized: glutathione peroxidase (GPX), plasma selenoprotein P (SELENOP), phospholipid hydroperoxide glutathione peroxidase (GPX4), plasma GPX3, and thioredoxin reductase (TXNRD) (7). Cloning and expression of UGA-containing transcripts has now demonstrated that the mammalian selenoproteome consists of 24–25 selenoproteins (8, 9).

When these studies were conducted there was considerable interest in the optimum form of Se for supplementation and prevention of cancer (10–12). Both inorganic Se, like selenite, and organic Se, like selenomethionine (SeMet), had been shown to readily provide Se for GPX synthesis (13) and to prevent cancer in animal models (14). Some studies suggested that one form vs. another had differential bioavailability (15) or ability to prevent cancer (11). Dietary methionine (Met) had been shown to modulate Se incorporation from SeMet into GPX (13, 16, 17) and to prevent mammary tumors (18) because, unlike inorganic forms, intact SeMet is an excellent analog of Met for incorporation into general body proteins in place of Met, thus raising tissue Se content without biochemical activity (16, 17, 19). Selenobetaine (SeBetaine) as a methylselenol donor has high potency against DMBA-induced mammary tumors but it was postulated that anticarcinogenic effects of SeBetaine might be exerted without metabolism to selenoproteins (20). Se was also known to be toxic at higher levels (21–23), but it was not clear if there were additional selenoproteins that appear only under high Se status or that are associated just with Se toxicity (24, 25).

Thus, we developed a procedure using SDS slab gel gradient electrophoresis (SDS/PAGE) that separates and quantitates the various Se-containing protein subunits, including GPX (26). By sacrificing rats at various times after the iv injection of Se into rats, SDS/PAGE can monitor the flux of radioactive Se into and between the various detected selenoproteins. As reported previously only in abstract form, we used this procedure to examine the incorporation of ^75^Se from selenite (27), selenodiglutathione (28), selenate (29), selenomethionine (30), and SeBetaine (31) in order to study Se metabolism leading to selenoprotein synthesis.

The prevailing thought at the time was that tissue Se is present as Sec in selenoproteins, as SeMet is incorporated into general body proteins, and as low molecular weight (MW) metabolites such as selenide, glutathione-Se intermediates, and methylated forms such as methylselenol (7). Low MW “selenosugar” species – seleno-N-acetyl galactose amine (SeGalNac) – first found in urine has also been found in liver as CH_3_-SeGalNac and GS-SeGalNac (32). Note that the Se in these selenosugars is linked to galactose 1-carbon via a Se-C bond. Using HPLC coupled with Se-specific and molecule-specific mass spectroscopy, we recently found these low-MW species in livers of turkeys fed Se-adequate and high-Se diets, but we also found high-MW selenosugar species linked via selenodisulfide bonds (Se-S) to protein. Surprisingly, more Se was present as the selenosugar moiety in Se-adequate turkey liver, mostly decorating general proteins, than was present as Sec in selenoproteins; with high Se supplementation, these “selenosugar-decorated” proteins were further increased (33). This study on turkey liver shows the power of these approaches and more modern analytical techniques to uncover the full metabolism of Se.

Our hypotheses at the time were that ^75^Se from injected ^75^Se selenocompounds would be distributed differently in rats, would result in different ^75^Se-labeling patterns of selenoproteins, and might lead to novel ^75^Se-labeled selenoproteins under high Se status. We found, however, that there were no dramatic differences in ^75^Se distribution between tissues, and that these selenocompounds were not differentially or preferentially metabolized to provide Se for selenoprotein incorporation. SDS/PAGE also did not detect ^75^Se-labeling of novel selenoproteins under high Se status. What we did not recognize then was the importance of tissue ^75^Se that was missing from the SDS/PAGE gels.

## Materials and Methods

### Rat Procedures

The series of studies reported here were conducted in 1986–1991 and approved by the following Animal Care and Use Committees: University of Arizona (A3248 #86-0172 and #86-0357), and the University of Missouri (A3394 #1425). Male Holtzmann weanling rats were fed a basal 30% torula yeast-based diet that contained by analysis 0.005–0.018 μg Se/g diet (26, 34, 35). To prevent liver necrosis, the basal diet was supplemented with 100 IU/kg of all rac-α-tocopheryl acetate (Sigma Chemical Co., St. Louis, MO) at the expense of sucrose. Unless otherwise stated, the basal diet was supplemented with 0.4% D,L-methionine (U.S. Biochemical Corp., Cleveland, OH), and with 0, 0.2, 2.0, and/or 5.0 μg Se/g diet as selenite for 50–80 d, depending on the experiment. Rats were anesthetized with ether and injected iv in the femoral vein with 50 μCi of ^75^Se-labeled selenocompounds at trace levels (~0.5 μg Se), and killed 1, 3, 24, or 72 h (also 168 h for SeMet) after injection as described previously (26). Blood was sampled by cardiac puncture using a heparinized syringe; liver was perfused *in situ* with 0.15 M KCl to remove erythrocytes. Plasma was obtained by centrifugation (1,000 g × 30 min). Tissues were weighed, and portions of tissues were ^75^Se-counted to calculate tissue ^75^Se recovery. Liver and kidney were homogenized in 9 vol of 0.25 M sucrose, and the cytosolic fractions were prepared by subcellular fractionation. Heart, testes, and muscle (gastrocnemius from uninjected (right) leg) were homogenized in 9 vol of 10 mM Tris, 1% SDS, and 10 mM 2-mercaptoethanol buffer, pH 7.4, using a Brinkmann polytron, and the homogenates were centrifuged at 105,000 g x 60 min to obtain supernatants that were then subjected to SDS/PAGE (26).

### SDS/PAGE Procedure

After preparation, 1,500 μg protein was mixed (1:1) with sample buffer (50 mM Tris, 1% SDS, 2% 2-mercaptoethanol), heated in a boiling water bath for 15 min, and loaded onto 3 mm slab gels with an acrylamide gradient from 7.5 to 20% (top to bottom) and electrophoresed at 60 mA per gel. The gels were fixed in methanol:acetic acid:water (5:1:4) containing 0.25% Coomassie brilliant blue R, and destained in methanol:acetic acid:water (75:50:875). Each lane was cut out, sliced into 2 mm slices, and counted. Protein standards of known MW were run to calibrate position with molecular weight (26).

### ^75^Se Compounds

[^75^Se]selenite was obtained from commercial sources or produced at the Research Reactor at the University of Missouri. Individual rats were injected with 50 μCi of [^75^Se]selenite (~0.5 μg Se). L-[^75^Se]SeMet (1.1 Ci/μmole) was obtained from Amersham. [^75^Se]selenodiglutathione was synthesized from 2 mCi of [^75^Se]selenite (63 μCi/μg Se), which were reduced with 5 mg ascorbate, oxidized with concentrated redistilled HN0_3_ and then dried at 60oC. The resulting selenite (0.24 μmoles) was reduced with GSH on ice for 16 h at pH < 1.0, using a 4 GSH:1 Se stoichiometry. A sample was analyzed using a Dowex-Ni column, which showed that 97% of the applied ^75^Se eluted after GSSG and thus was present as [^75^Se]GSSeSG (36). [^75^Se]selenobetaine (dimethylselenoacetate, SeBetaine) was synthesized from 2.5 mCi of [^75^Se]selenite (70 μCi/μg Se) by borohydride reduction, and reacted with iodomethane to produce trimethylselenonium ion. After purification, the trimethylselenonium ion was pyrolyzed to form dimethylselenide, which was reacted with bromoacetic acid to form dimethylselenoacetate. Purification on SP-Sephadex resulted in >60% recovery as SeBetaine (37). [^75^Se]selenate was prepared by oxidizing [^75^Se]selenite with 30% H_2_O_2_. Following oxidation, complexation with 2, 3- diaminonaphthalene, a selenite-specific reaction, showed that <3% of the ^75^Se remained as selenite. A Packard model 5650 refrigerated gamma counter with 3” KI crystal was used for ^75^Se counting (60% efficiency for ^75^Se).

## Results

Biomarkers of Se status of Holtzmann rats fed these diets have been reported thoroughly by our group and are not reported here. Plasma and liver glutathione peroxidase activities in rats fed the Se-deficient basal diet are typically 2% of levels found in rats supplemented with 0.2 μg Se/g diet (35) and are not further increased by 1–5 μg Se/g diet (35, 38). Liver Se concentrations for rats fed the basal Se-deficient diets for 4 wk are typically 0.25 nmol/g liver (0.02 μg Se/g) and 3% of levels in rats fed 0.2 μg Se/g diet. Liver Se concentration in rats fed 0.2, 2, and 5 μg Se/g are typically 0.66, 2.2, and 2.9 μg Se/g liver, respectively (38).

**Figure 1:**
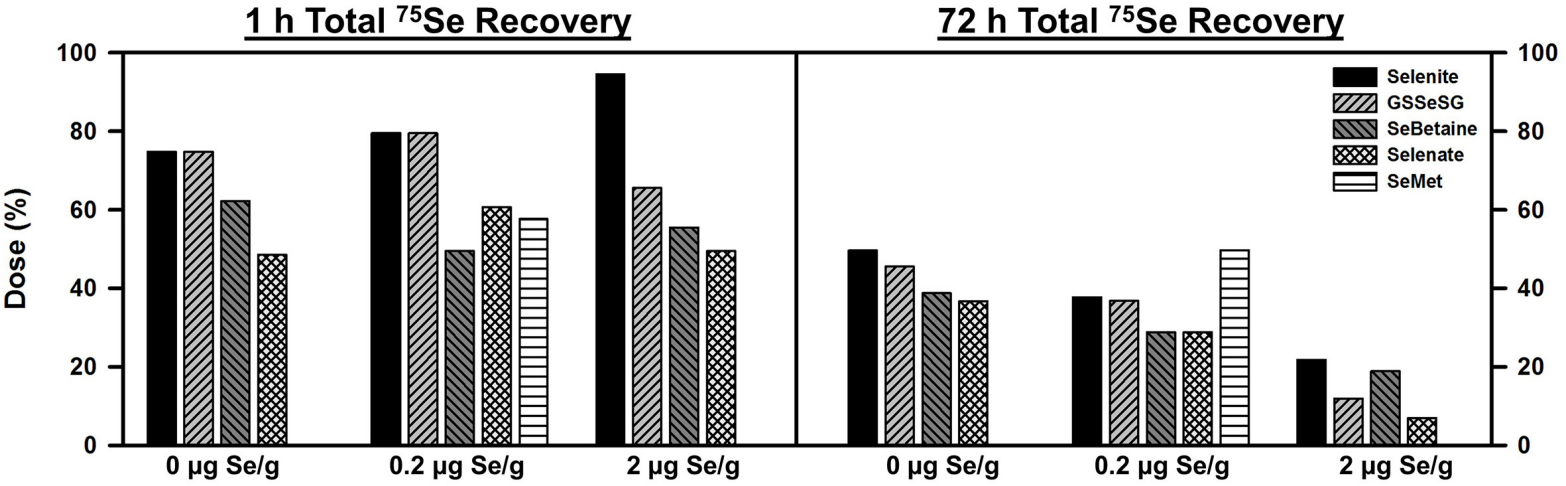
Total ^75^Se recovery at 1 and 72 h after injection ^75^Se selenocompounds. Rats were fed the indicated dietary Se levels as selenite for 50–80 d, injected iv with 50 μCi of tracer ^75^Se-labeled Selenite, GSSeSG, SeBetaine, Selenate, or SeMet, killed at 1 and 72 h afer dosing, and ^75^Se retained in blood, liver, kidney, heart, testes, epididymus, and muscle was counted. Values are the percent of the administered ^75^Se dose recovered (*n* = 1 per treatment at each time for each selenocompound, *n* = 26 total).

Se status did not have a large effect on ^75^Se recovery, distribution, or retention for any of the compounds tested (Figure 1). At 1 h, 75 to 50% of the injected ^75^Se was recovered in blood, liver, kidney, heart, muscle, and testes of these male rats, regardless of the form of Se. Selenite and GSSeSG recoveries were slightly higher than for SeBetaine, selenate, and SeMet at 1 h. By 72 h, total retention in these tissues ranged from 50 to 25% when injected into 0 and 0.2 μg Se/g diet rats, but <20% for rats fed 2 μg Se/g. At 72 h, total retention in rats fed 5 μg Se/g as selenite was 11%. ^75^Se recovery from SeMet was only determined in rats fed 0.2 μg Se/g, but appeared higher than for the other forms. For the other four Se compounds, the recoveries at 72 h in rats fed 0.2 vs. 0 μg Se/g were only marginally reduced, as compared to the decrease in dietary Se concentration, suggesting that the relative flux of Se in rats fed 0.2 μg Se/g was little altered as compared to rats fed the Se-deficient diet.

**Figure 2:**
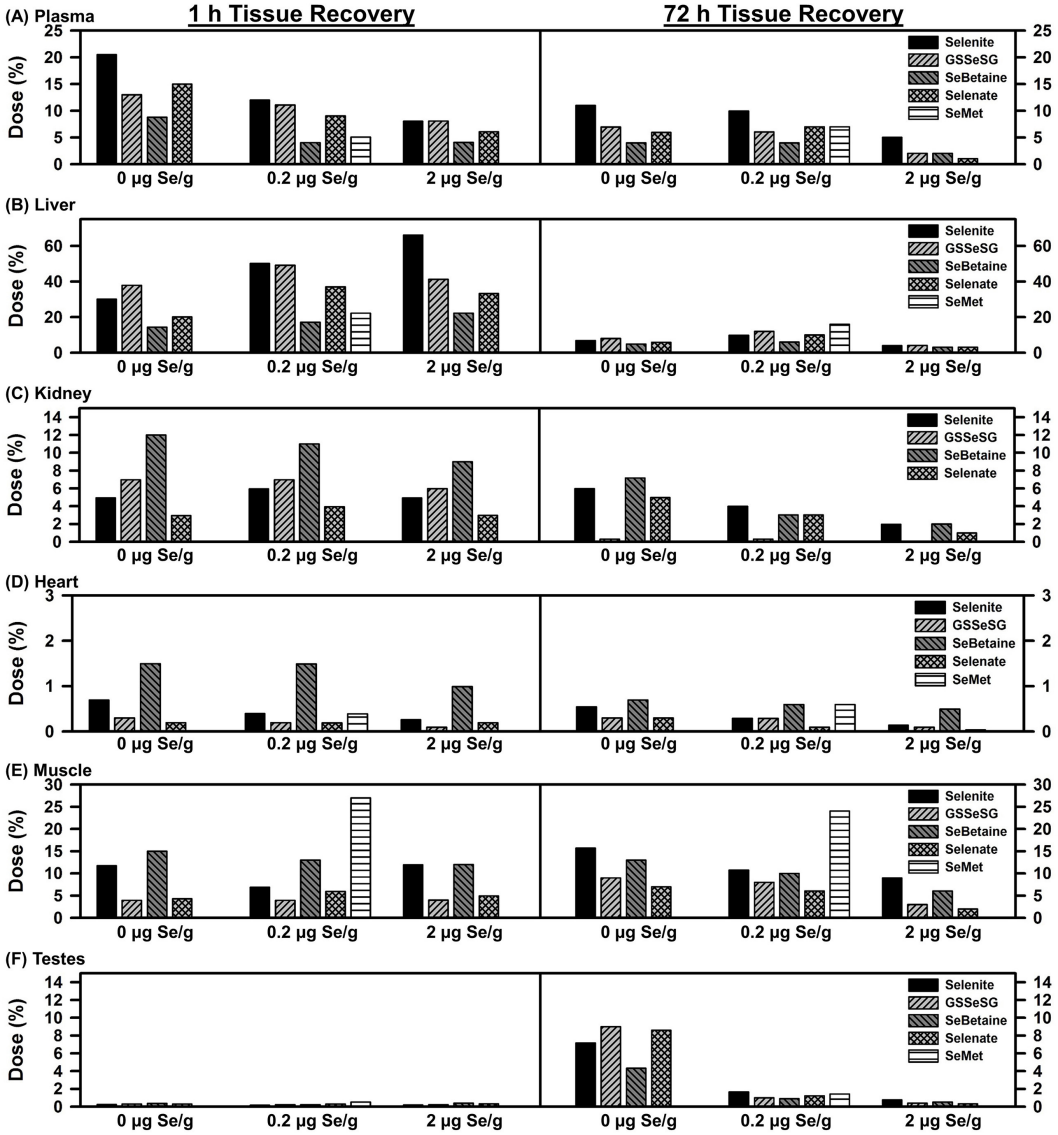
Tissue ^75^Se recovery at 1 and 72 h after injection ^75^Se selenocompounds. Rats were treated as described for Figure 1. Values are the percent of the administered ^75^Se dose recovered in each tissue (*n* = 1 per treatment at each time for each selenocompound, *n* = 26 total).

Recovery of injected ^75^Se in six tissues are shown in Figure 2. Plasma ^75^Se retention was calculated based on a blood volume of 8% of total body weight and fraction of blood as plasma (26). At 1 h, 20% of the injected ^75^Se was found in plasma in rats fed the Se-deficient diet. Supplemental dietary Se at 0.2 and 2 μg Se/g diet progressively deceased the recovered ^75^Se in plasma. By 72 h, plasma retained ~10% of the [^75^Se]selenite in rats fed both 0 and 0.2 μg Se/g diet, but this was decreased to 5% with 2 μg Se/g diet. At 72 h, approximately half as much injected ^75^Se was retained for the other selenocompounds as compared to selenite.

A different pattern was found for liver as compared to plasma. At 1 h, recovery of ^75^Se from selenite doubled in rats fed 2 vs. 0 μg Se/g, and recoveries of ^75^Se from GSSeSG, SeBetaine, and selenate were the same or higher in rats fed 0.2 and 2 μg Se/g diet as compared to rats fed the Se-deficient diet, suggesting a greater flux of ^75^Se to other tissues in Se-deficient rats. By 72 h, recoveries were 4% or less in liver for all selenocompounds regardless of dietary Se; in rats fed 5 μg Se/g as selenite, liver ^75^Se recoveries were 59 and 3% at 1 and 72 h, respectively, similar to levels in rats fed 2 μg Se/g (data not shown). Overall, there was little effect of Se status on the recovery of ^75^Se in liver.

Kidney, however, provided a third pattern. The level of dietary Se supplementation had little effect on recovery of ^75^Se at 1 h. Furthermore, the recovery of ^75^Se at 1 h especially for SeBetaine but also GSSeSG was higher than for selenite, reflecting either targeted uptake by kidney, or reduced uptake/retention by liver and plasma. By 72 h, there was little ^75^Se arising from GSSeSG found in kidney.

Heart also displayed higher retention of ^75^Se from SeBetaine as compared to the other Se compounds at 1 h. And relative to plasma, liver, and kidney, retention of ^75^Se at 72 h in heart remained more similar to retention levels at 1 h.

Recovery of ^75^Se in muscle was calculated estimating that muscle was 40% of the total body weight of the rat (26). Recovery of ^75^Se at 1 h and 72 h were almost identical for all Se compounds, and little affected by level of dietary Se. Even at 1 h, SeMet ^75^Se retention was 4-times the level of selenite ^75^Se retention in rats fed 0.2 μg Se/g. This distribution clearly shows the specific uptake and retention of SeMet relative to the other injected selenocompounds.

At 1 h, there was almost no ^75^Se found in testes regardless of the form of Se administered. By 72 h, testes in Se-deficient rats retained 4–8% of the administered ^75^Se. ^75^Se retention was dramatically reduced in rats fed 0.2 μg Se/g diet, and further reduced in rats fed 2 μg Se/g. In contrast to the other five tissues, injected ^75^Se was targeted to testes in Se deficiency, but this targeted flux was curtailed in Se-adequate male rats.

### SDS/PAGE Analysis

The use of the SDS/PAGE analysis of ^75^Se incorporation into selenoproteins used 2-mercaptoethanol treatment to separate protein subunits according to MW, and to reduce “loosely bound Se from proteins.” Mercaptoethanol treatment will also reduce selenodisulfide linkages, thus releasing low-MW Se forms bound to proteins through these links. Subsequent SDS/PAGE eluted resulting low-MW species into the bottom buffer so that the resulting profiles only display high-MW protein subunits containing Sec. Potentially also retained on the gel might be other high-MW proteins with Se-C bonds, but this would not include Se species linked via selenodisulfide linkages such as selenosugars linked to protein cysteines. The result is the clean profiles of selenoproteins we reported in 1988 as compared to the gel filtration profiles, which showed 4 broad peaks, including >250 kDa species at the void volume, the ~100 kDa peak containing tetrameric GPX1, the ~20 kDa peak containing GPX4, and the largest peak containing low-MW species eluting at the column volume (26). Follow-up SDS/PAGE analysis of these individual peaks showed that the 100 kDa peak contained 23 kDa GPX1 subunits and the 20 kDa peak contained GPX4 polypeptide; the >250 kDa and low-MW peaks contained no ^75^Se-labeled protein peaks after this 2-mercaptoethanol + SDS/PAGE analysis (39). These ^75^Se species are the “missing” selenometabolites not detected in our use of SDS/PAGE to analyze for selenoproteins.

**Figure 3:**
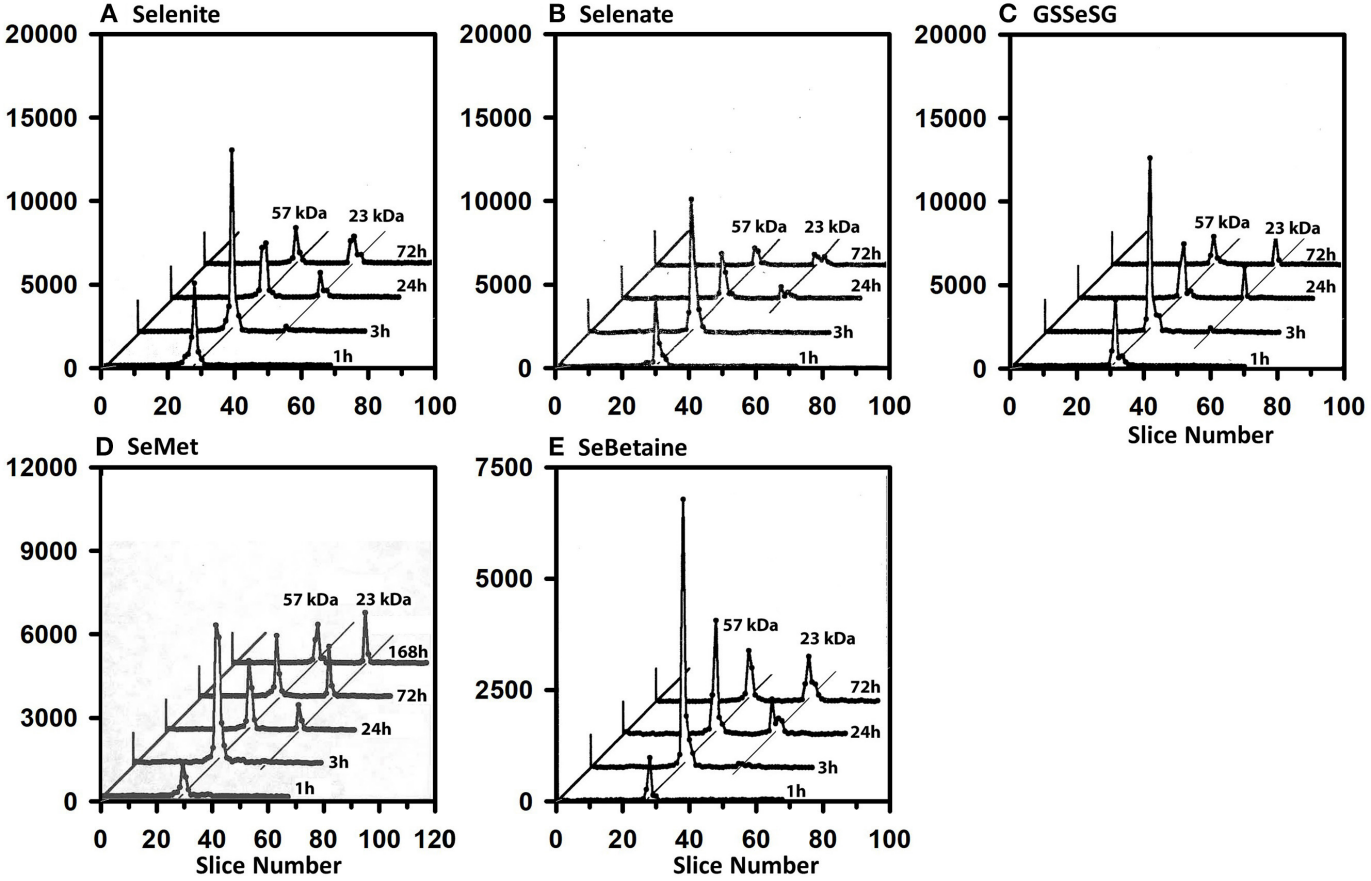
Incorporation of ^75^Se into plasma proteins. Rats were fed 0.2 μg Se/g diet as selenite for 50–80 d, injected iv with 50 μCi of tracer ^75^Se-labeled Selenite **(A)**, Selenate **(B)**, GSSeSG **(C)**, SeMet **(D)**, and SeBetaine **(E)**, and killed at 1, 3, 24, or 72 h (or 168 h for SeMet) afer dosing. Plasma samples (1,500 μg protein) were separated using gradient SDS/PAGE. Sample lanes in each gel were cut into 2-mm slices and counted. The cpm for each slice are plotted to show ^75^Se incorporation into selenoproteins of different subunit molecular weights. Slice 1 contains polypeptides of the highest molecular weight. Plots for 3, 24, and 72 h were staggered, and diagonal lines are drawn through the major ^75^Se proteins to show the change in ^75^Se incorporation in these species with time. Each time profile in each selenocompound panel is from one rat (*n* = 21 total rats). Plasma SELENOP is 57 kDa and GPX3 is 23 kDa.

Full-length plasma SELENOP has a peptide MW of 43 kDa but is glycosylated to have an apparent MW of 57 kDa (40). Figure 3 shows the SDS/PAGE ^75^Se profile in plasma for the five selenocompounds at 1, 3, 24, and 72 h after iv ^75^Se injection in rats fed 0.2 μg Se/g as selenite. The profiles are all remarkably the same. Maximum incorporation into SELENOP is observed at 3 h as reported previously (40). Notable ^75^Se incorporation into plasma GPX3 is not observed until 24 h, and this level of incorporation remains at 72 h. These profiles clearly indicate that all five selenocompounds are rapidly metabolized to the common precursor used for incorporation into selenoproteins.

**Figure 4:**
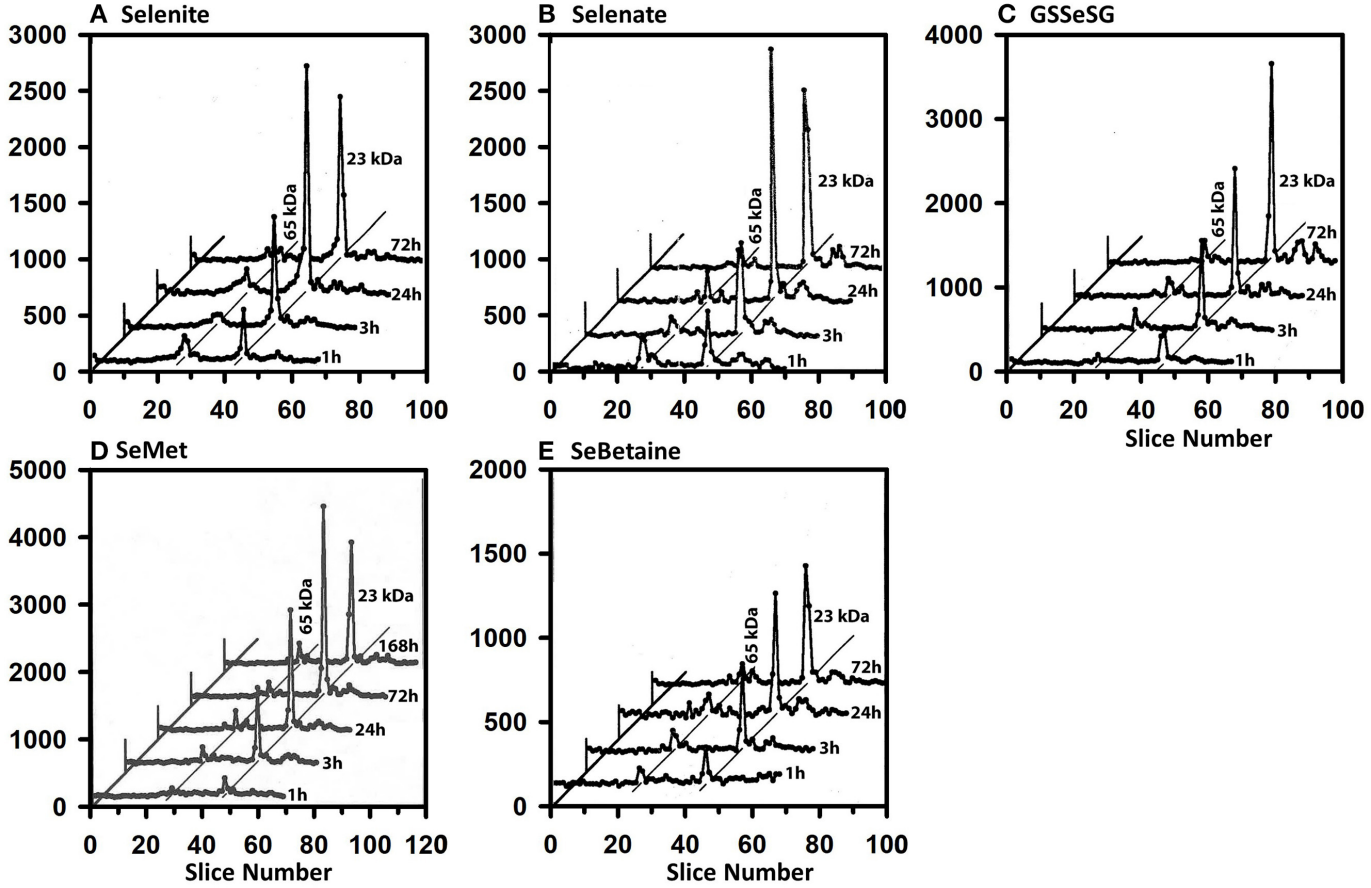
Incorporation of ^75^Se into liver cytosolic proteins from Selenite **(A)**, Selenate **(B)**, GSSeSG **(C)**, SeMet **(D)**, and SeBetaine **(E)**. Rats were treated as described for Figure 3. After homogenization and centrifugation at 105,000 g × 60 min, cytosols (1,500 μg protein) were separated using gradient SDS/PAGE as described for Figure 3 (*n* = 21 total rats). Liver GPX1 is 23 kDa, and the 65 kDa species are likely to the isoenzymes of TXNRD1.

The ^75^Se profiles in liver are also all remarkably the same for the five compounds (Figure 4). By 1 h, substantial injected ^75^Se was rapidly incorporated into the 23 kDa GPX1 subunit, with maximal ^75^Se labeling with selenite and selenate at 24 h. At 72 h, ^75^Se incorporation into GPX1 from GSSeSG and SeMet was even higher than at 24 h, suggesting these species were more slowly metabolized into the Se precursor than for selenite and selenate. The reduced uptake of ^75^Se from SeBetaine into liver resulted in slower labeling of GPX1. In addition, several additional selenoprotein subunits of 65 and 19 kDa were also labeled, but at far lower levels than for GPX1. These species are likely to be cytosolic thioredoxin reductase 1 (TXNRD1) with isoforms at 63 and 55 kDa, and GPX4 at 19 kDa.

**Figure 5:**
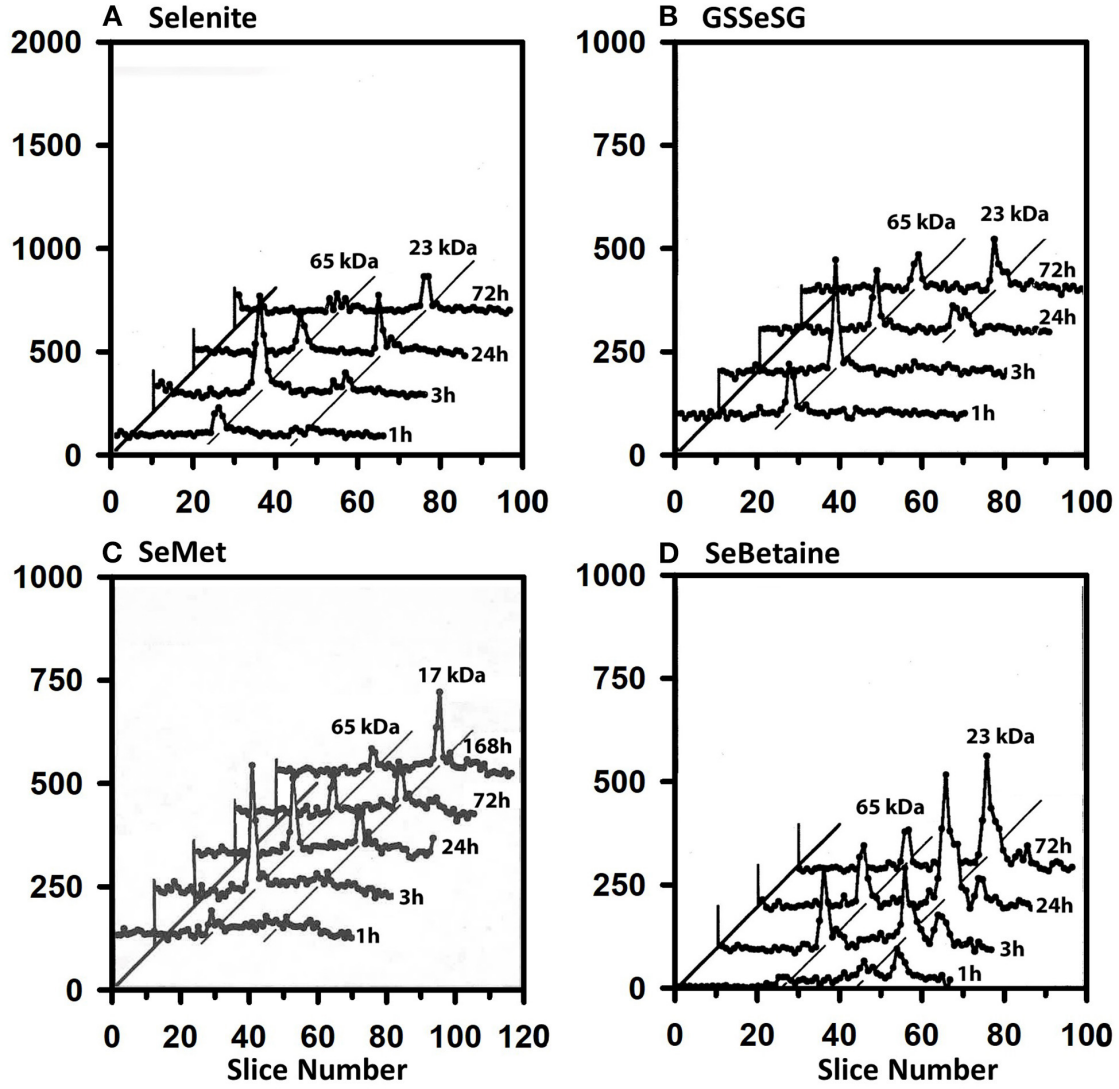
Incorporation of ^75^Se into heart supernatant proteins from Selenite **(A)**, GSSeSG **(B)**, SeMet **(C)**, and SeBetaine **(D)**. Rats were treated as described for Figure 3. Heart tissue was homogenized in Tris/SDS and centrifuged at 105,000 x g, and supernatants (1,500 μg protein) were separated using gradient SDS/PAGE as described for Figure 3 (*n* = 17 total rats). GPX1 is 23 kDa, and the 65 kDa species are likely to the isoenzymes of TXNRD.

The ^75^Se selenoprotein profiles of heart supernatant (Figure 5) are also very similar for all four selenocompounds. At 1 and 3 h, the 65 kDa species contained more ^75^Se than in GPX1 subunits for selenite, GSSeSG, and SeMet. This labeling diminished somewhat by 24 h when GPX1 subunit gained prominence, but both species retained ^75^Se labeling at 72 h.

**Figure 6:**
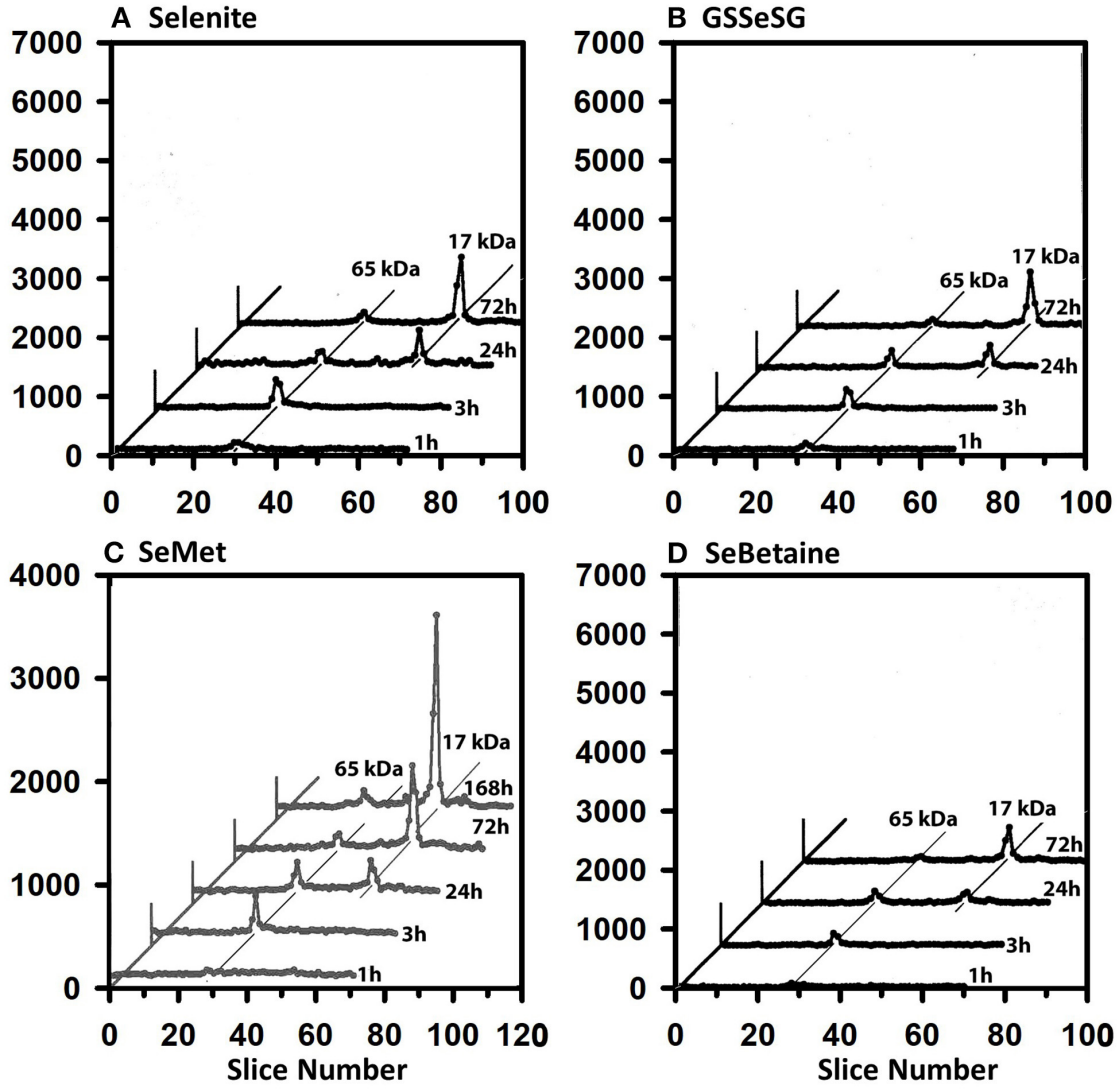
Incorporation of ^75^Se into testes supernatant proteins from Selenite **(A)**, GSSeSG **(B)**, SeMet **(C)**, and SeBetaine **(D)**. Rats were treated as described for Figure 3. Testes supernatants were prepared and analyzed by SDS/PAGE, as described for Figure 5 (*n* = 17 total rats). GPX4 is 17 kDa, and the 65 kDa species are likely to the isoenzymes of TXNRD.

The ^75^Se profiles of testes supernatant show a different story (Figure 6). Early on, the 65 kDa species were labeled at 3 h, but by 24 h the 17 kDa GPX4 is equally ^75^Se-labeled from selenite, GSSeSG, SeMet, and SeBetaine. The GPX4 was the dominate ^75^Se-labeled selenoprotein at 72 h.

### Effect of Se Status on ^75^Se-Selenoprotein Labeling

**Figure 7:**
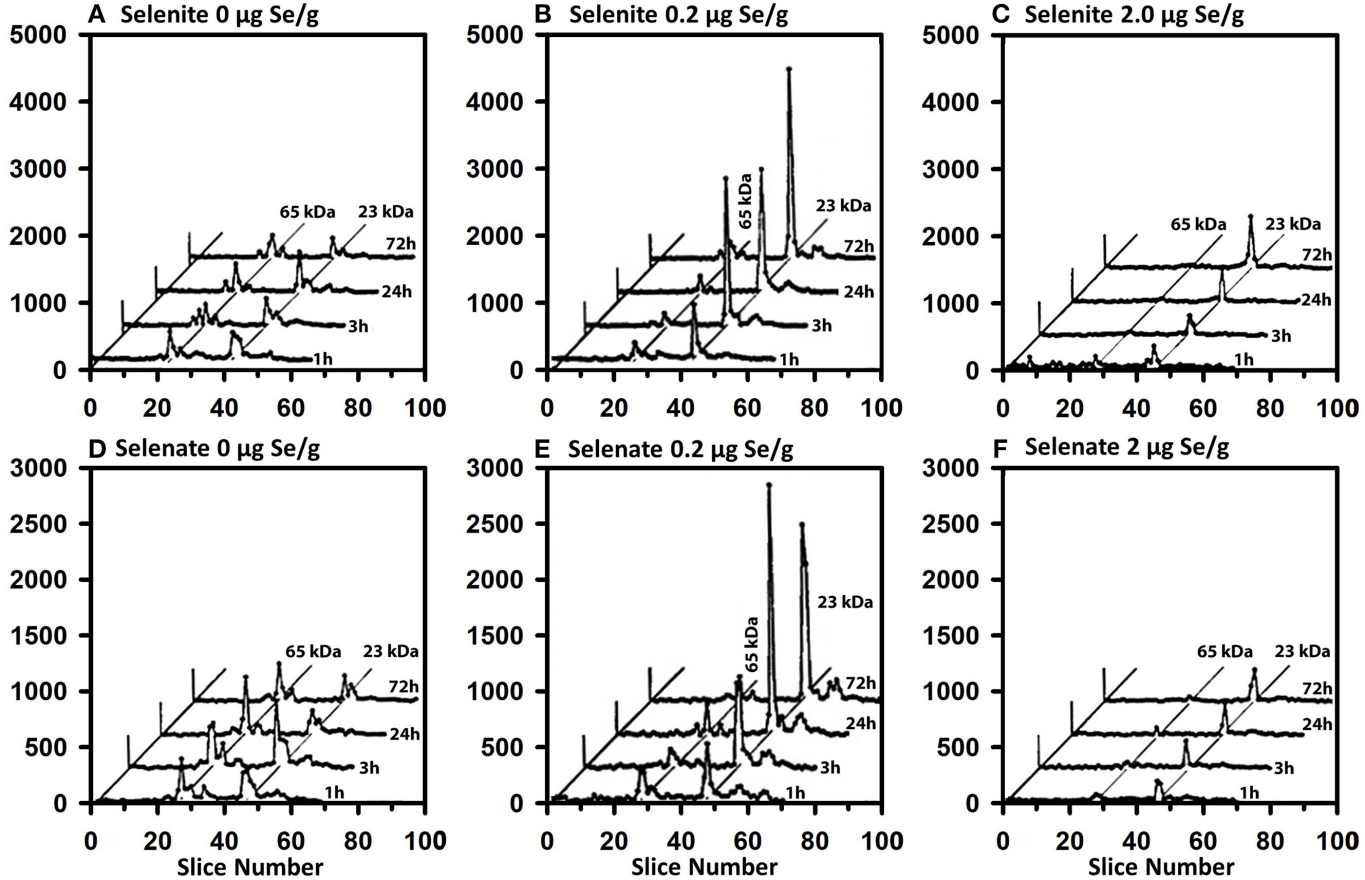
Incorporation of ^75^Se from [^75^Se]selenite **(A–C)** and [^75^Se]selenate **(D–F)** into liver cytosolic proteins. Rats were fed the indicated dietary Se levels as selenite for 50–80 d, injected iv with 50 μCi of tracer ^75^Se-labeled selenite or selenate, and killed at 1, 3, 24, or 72 h afer dosing. After homogenization and centrifugation at 105,000 x g, cytosols (1,500 μg protein) were separated using gradient SDS/PAGE as described for Figure 3 (*n* = 24 total rats). Liver GPX1 is 23 kDa, and the 65 kDa species are similar to the isoenzymes of TXNRD1.

The same-sized 50 μCi tracer dose of ^75^Se was injected at various times into rats fed Se-deficient (0 μg Se/g diet), Se-adequate (0.2 μg Se/g diet), and high Se (2 μg Se/g diet) to study the impact of Se status on flux of ^75^Se into liver selenoproteins (Figures 7A–C). In Se-deficient liver, there was little effect of time after dosing on incorporation into selenoproteins, in contrast to what was observed in Se-adequate rats (Figure 4). Furthermore, in Se-deficient rats, the amount of ^75^Se labeling of GPX1 was the same as the labeling of TXNRD at all times (Figure 7A), whereas ^75^Se labeling of GPX1 increased dramatically in Se-adequate liver from 1 to 3 to 24 h after dosing (Figure 7B). In high-Se rat liver, there was little incorporation of ^75^Se into the 65 kDa species; ^75^Se incorporation into GPX1 was considerably less as compared to Se-adequate liver, with the more modest incorporation doubling from 3 to 24 h, and doubling again from 24 to 72 h. The pattern of ^75^Se incorporation from [^75^Se] selenate (Figures 7D–F) was virtually the same as that observed with [^75^Se]selenite, showing that both selenocompounds are metabolized in intact rats at similar rates into the precursor used for Se incorporation into selenoproteins.

### Effect of Met Status on ^75^Se-Selenoprotein Labeling From [^75^Se]SeMet

**Figure 8:**
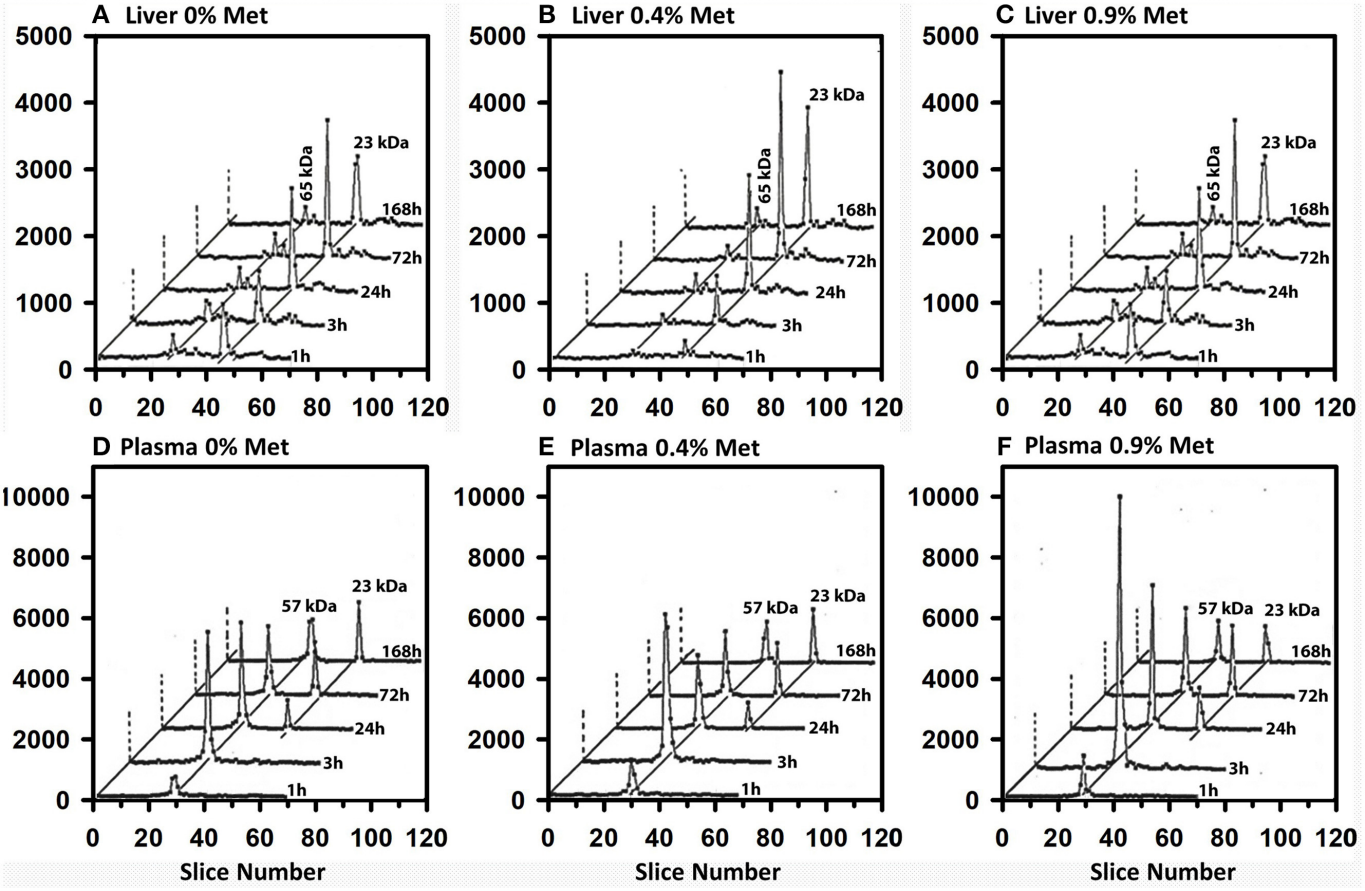
Incorporation of ^75^Se from [^75^Se]SeMet into liver cytosolic **(A–C)** and plasma **(D–F)** proteins. Rats were supplemented with 0.2 μg Se/g diet as selenite in the basal diet containing 0.4% supplemental Met for 50–80 d. For 7 days prior to Se injection, supplemental Met was adjusted to 0, 0.4 or 0.9% D,L-Met; rats were then injected iv with 50 μCi of tracer ^75^Se-labeled SeMet, and killed at 1, 3, 24, 72, or 168 h afer dosing. Liver cytosol and plasma samples were prepared as described in Figures 3 and 4 (*n* = 30 total rats). Liver GPX1 is 23 kDa, and the 65 kDa species are similar to the isoenzymes of TXNRD1; plasma SELENOP is 57 kDa and GPX3 is 23 kDa.

Because SeMet mixes with the Met pool and is incorporated non-specifically as a Met analog into general body proteins (16, 17, 19), we studied the effect of feeding three levels of dietary Met for 1 wk in the Se-adequate diet (0.2 μg Se/g diet as selenite) prior to injection of 50 μCi of tracer [^75^Se]SeMet. Without Met supplementation, ^75^Se incorporation into liver GPX1 from [^75^Se] SeMet was approximately half the level of incorporation from [^75^Se]selenite at all times in Se-adequate liver (Figures 8A–C). With 0.4% Met supplementation, the labeling of GPX1 from tracer [^75^Se]SeMet was similar to that from [^75^Se]selenite. Doubling dietary Met supplementation to 0.9% Met perhaps only slightly decreased the labeling of GPX1 relative to that observed with 0.4% dietary Met, suggesting that there was little enhanced release of ^75^Se from [^75^Se]SeMet to the precursor form of Se used for selenoprotein synthesis, at least in liver.

In contrast to liver, a different pattern of ^75^Se incorporation from [^75^Se]SeMet into plasma SELENOP was observed for the three levels of dietary Met (Figures 8D–F). The selenoprotein labeling patterns for SELENOP and GPX3 were virtually the same when Se-adequate rats were supplemented with 0 or 0.4% dietary Met for 1 week. Higher dietary Met supplementation at 0.9%, however, doubled the ^75^Se labeling of plasma SELENOP at 3 and 24 h, as compared to labeling in 0.4% Met rats, indicating that there was increase SeMet catabolism releasing ^75^Se for incorporation into SELENOP.

### The “Missing” ^75^Se

We used 2-mecaptoethanol treatment and SDS/PAGE analysis to focus on the flux of ^75^Se into true selenoproteins, with the presumption that this would strip away low-MW selenometabolites and loosely bound selenospecies, including species linked by disulfide bonds. Our recent finding that low-MW and high-MW selenosugars are present in high quantities in Se-adequate and high-Se liver at least in turkeys (33), however, strongly suggests that the ^75^Se we did not find in the SDS/PAGE ^75^Se profiles is also important.

**Figure 9:**
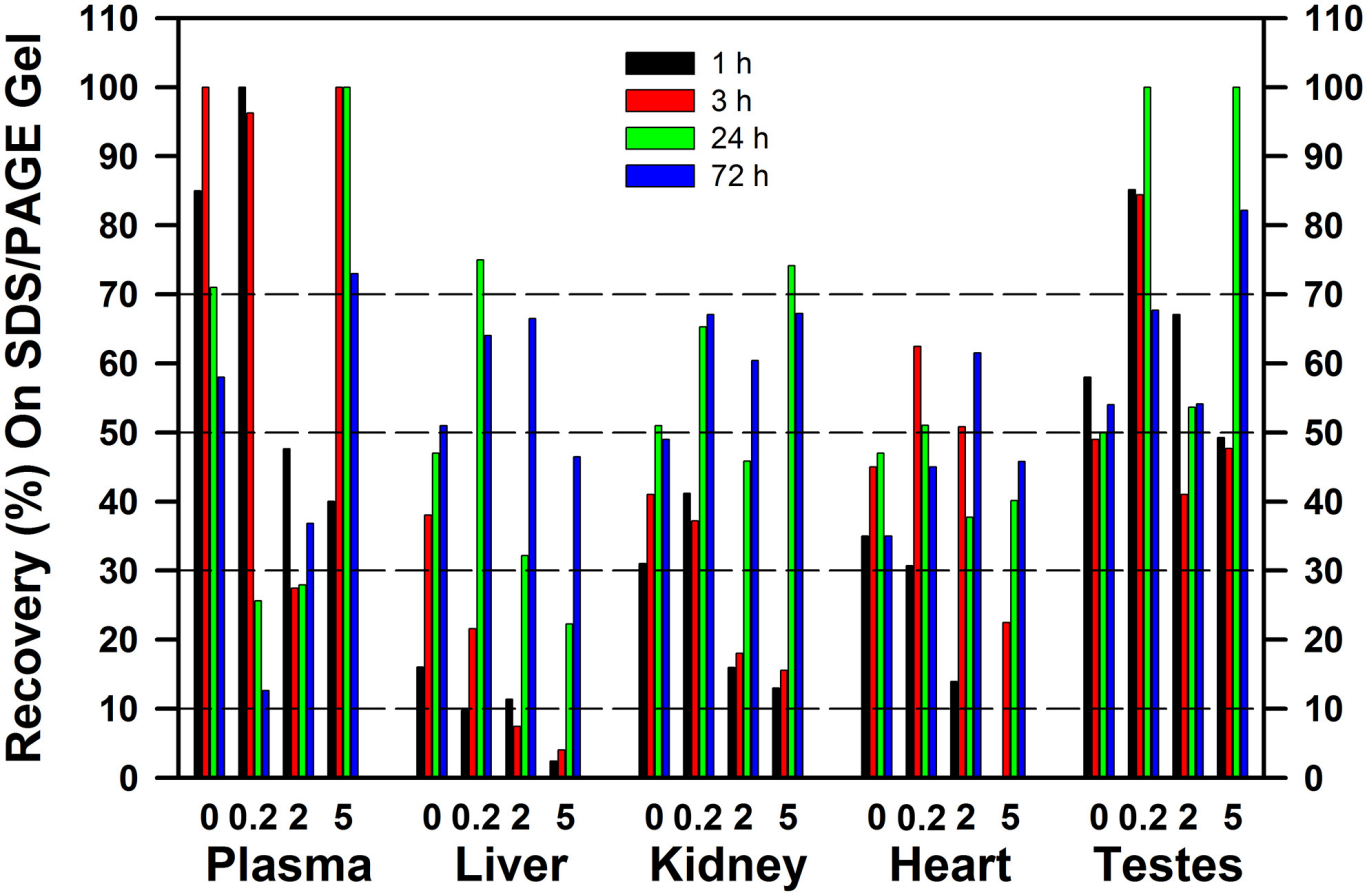
Recovery of ^75^Se from [^75^Se]selenite as ^75^Se-labeled selenoproteins on SDS/PAGE gels. Rats were supplemented with 0, 0.2, 2, or 5 μg Se/g diet as selenite for 50–80 d, injected iv with 50 μCi of [^75^Se]selenite, and were tissues subjected to SDS/PAGE as described for Figures 2–6 (*n* = 16 total rats). Values are the percent of the applied ^75^Se recovered in the gel after SDS/PAGE in the indicated tissues at the indicated times.

The recoveries of ^75^Se in the gels following [^75^Se]selenite injection are shown in Figure 9 for rats fed 0 to 5 μg Se/g as selenite. The major plasma selenoprotein (Figures 3, 8), SELENOP, is synthesized and secreted by the liver; the recovery of >70% of the applied ^75^Se in plasma as SELENOP at 1 and 3 h after injection agrees other reports (40). Similarly, recovery of >50% of the injected ^75^Se in the SDS/PAGE gels in testes supernatant, regardless of Se status, might be expected as SELENOP is synthesized predominately by the liver, secreted, and then specifically targeted to the testes as mediated by the APOER2 receptor (LRP8) (41). In liver at 1 h, however, <15% of the applied ^75^Se in liver cytosol was recovered on the gel as Sec-containing selenoproteins, regardless of Se status. At 3 h <40% was recovered in Se-deficient rat liver and <20% in Se-adequate rat liver; this low recovery matches with the lack of ^75^Se labeling of liver GPX1 at 1 and 3 h (Figures 4, 7). Increasing Se status decreased the recovery ^75^Se at both 1 and 3 h, such that <5% of the applied Se was recovered as Sec selenoproteins in liver cytosol from rats fed 5 μg Se/g. A similar effect of Se status was observed in kidney at 1 and 3 h, although the ^75^Se recovered in kidney was double the recoveries observed in liver. By 24 h in both liver and kidney, 30–50% of the applied ^75^Se was recovered in the gels, consistent with the increased labeling of GPX1. Similar patterns were observed for tracer studies providing ^75^Se as selenate or GSSeSG (data not shown). Clearly substantial cytosolic ^75^Se was present as species other than Sec in selenoproteins. Especially in liver, even at 24 and 72 h, there was a progressive decline in ^75^Se recovered as Sec selenoprotein as Se status increased from 0.2 to 2 to 5 μg Se/g diet.

**Figure 10:**
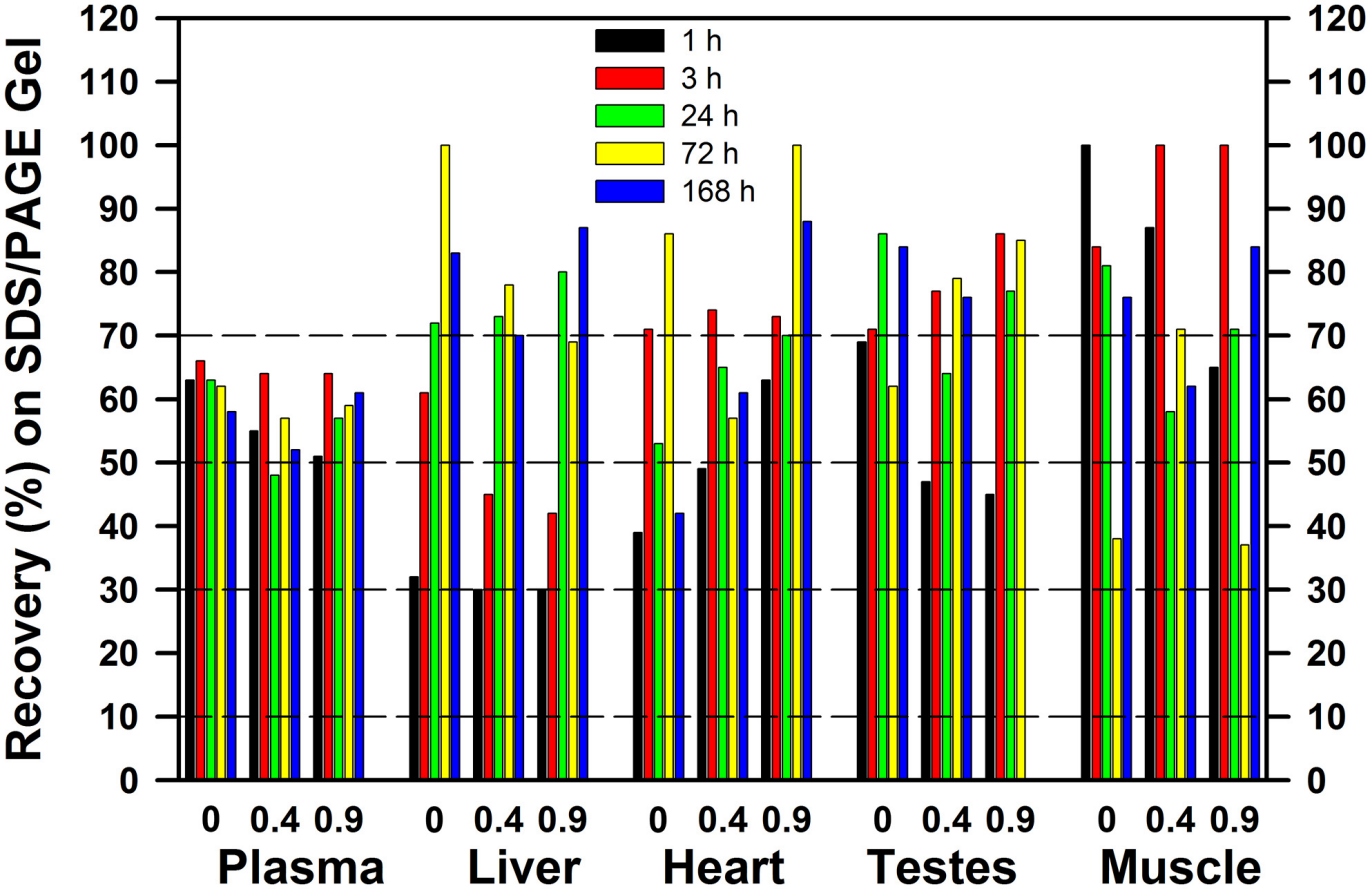
Recovery of ^75^Se from [^75^Se]SeMet as ^75^Se-labeled selenoproteins on SDS/PAGE gels. Rats were supplemented with 0.2 μg Se/g diet as selenite in the basal diet containing 0.4% supplemental Met for 50–80 d. For 7 days prior to Se injection, supplemental Met was adjusted to 0, 0.4, or 0.9% D,L-Met; rats were then injected iv with 50 μCi of [^75^Se]SeMet,and tissues were subjected to SDS/PAGE as described for Figures 2–6 (*n* = 15 total rats). Values are the percent of the applied ^75^Se recovered in the gel after SDS/PAGE in the indicated tissues at the indicated times.

When tracer ^75^Se was injected as SeMet, the patterns were very different (Figure 10), showing that the early fate of SeMet is decidedly different than for inorganic Se. At least 30% of the applied ^75^Se was recovered in the gel, regardless of tissue. With increasing time, there appears to be increased recovery of ^75^Se in as Sec in selenoproteins in liver and heart, but not in plasma. Feeding a marginal Met diet or doubling the diet Met, however, had little effect on incorporation of ^75^Se from SeMet into protein as assessed by recovery upon SDS/PAGE analysis. This matches with the selenoprotein profiles shown in Figure 8, with little effect of level of dietary methionine on the ^75^Se labeling of GPX1 in liver and SELENOP in plasma.

## Discussion

These studies used only adult Holtzmann rats from our colony that were fed the basal Se-deficient diets supplemented with graded levels of Se as selenite for 50–80 days. The data for an individual selenocompound at each time in these figures was only collected for a single rat, so only the resulting patterns can be compared. No statistical analysis was conducted.

Collectively, the studies reported here present data from 80 individual rats. The SDS/PAGE profiles for these ^75^Se tracer studies are very consistent and illustrate a constant time-driven pattern of ^75^Se corporation into selenoproteins in four tissues. The overall result is a clear pattern of very rapid Se metabolism from a diverse set of selenocompounds to a common intermediate used for synthesis and incorporation into well-defined ^75^Se selenoprotein patterns for at least the major selenoproteins: plasma SELENOP and GPX3, liver and heart GPX1 and the 65 kDa species (most likely TXNRD1), and testes GPX4. No selenocompound resulted in incorporation into a profoundly different set of at least these major selenoproteins. Even SeBetaine, which had been identified as having distinct activity to prevent DMBA-induced mammary tumors, resulted in these same patterns. SeMet was similarly rapidly metabolized to the precursor used for selenoprotein synthesis. Collectively, these studies emphasize that this wide variety of selenocompounds are not uniquely or preferentially metabolized to provide Se for selenoprotein incorporation.

**Figure 11:**
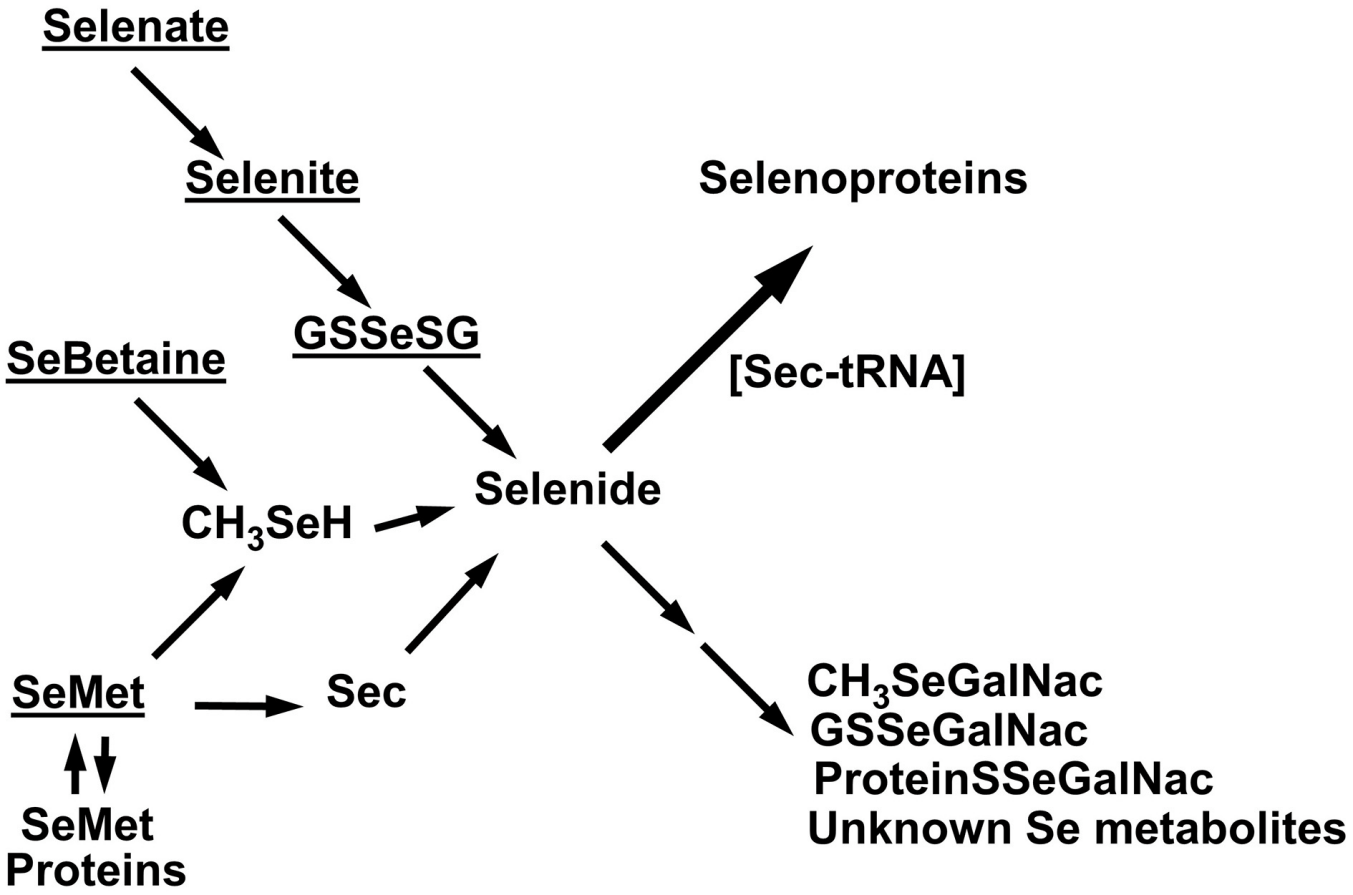
Schematic diagram of Se metabolism to selenoproteins. Underlined are the five tracer selenocompounds administered in these experiments. Selenate is reduced to selenite; selenite can react with GSH to form GSSeSG, which is then further reduced to selenide. SeMet is degraded via transulfuration to Sec which can be metabolized to selenide, or SeMet is degraded via transamination to methaneselenol and then selenide. Alternatively, SeMet can be incorporated into general body proteins as a methionine analog. SeBetaine is degraded to release methaneselenol. Selenide is the precursor used for selenoprotein incorporation, as assessed by SDS/PAGE in these studies. Metabolites not detected by SDS/PAGE include the low MW selenosugars, high-MW “selenosugar-decorated” proteins, and other unknown metabolites.

A schematic diagram of the metabolism of the five selenocompounds in these experiments is shown in Figure 11. All five ^75^Se tracers were readily and rapidly metabolized to the selenide-level precursor used for co-translational incorporation of Se as Sec into selenoproteins (1–6). The various pathways shown in Figure 11 have been discussed in detail previously (1, 3, 7, 20), with this same selenide-level selenospecies, the precursor for selenosugar synthesis (32, 33). The missing ^75^Se metabolites, not detected by SDS/PAGE as selenoproteins, include low-MW selenosugars, high-MW “selenosugar-decorated” proteins, and other unknown metabolites (32, 33).

These studies used tracer levels of ^75^Se. Estimates of total body burden of Se in an Se-adequate rat range from 48 to 61 μg total Se fed selenite (7, 42). Rats of this age consume ~30 g diet/d, so feeding 0.2 μg Se/g diet would provide ~6 μg of oral Se per day. A single injection of ~0.5 μg Se in rats fed 0.2 μg Se/g diet represents ~10% of the daily Se intake and ~1% of the total body burden of Se, and thus can be considered a tracer. In Se-deficient rats fed the 0.02 μg Se/g diet or ~0.6 μg per day, the 0.5 μg Se injection may represent an amount equivalent to that consumed in the diet. In a study with rats fed the Se-deficient diet for 60 d, injection of 15 μg Se as selenite failed to significantly raise GPX1 activity after 24 h (43). Here, the failure of the tracer ^75^Se injections to increase ^75^Se incorporation into GPX1 suggests the 0.5 μg Se dose was insufficient to substantially raise liver *Gpx*1 mRNA levels (Figure 7), further indicating that these were ^75^Se tracer studies even in Se-deficient rats.

It is thought that selenide or a GSH-selenide intermediate are the precursor species used in the first step in Sec synthesis (3, 6). These studies show that both the inorganic and the organic selenocompounds were rapidly metabolized to the Sec-synthesis precursor. Furthermore, the pattern and timing of ^75^Se labeling were almost identical in each tissue for all the selenocompounds. There was no apparent unique metabolism of one of these selenocompounds relative to the others, indicating that once internalized, the systemic Se metabolism of these species is the same. Furthermore, the similar timing for Se incorporation from these species indicates that the rate-limiting steps in selenoproteins synthesis occur after uptake and initial metabolism and are not associated with the differences in initial metabolism.

Today's understanding of selenoprotein expression and regulation can explain the observed ^75^Se labeling patterns. When tracer [^75^Se]selenite and [^75^Se]selenate were injected into Se-deficient rats, Se deficiency dramatically decreased the labeling of GPX1 in liver relative to Se-adequate rats at 3 to 72 h after injection, but had little effect on labeling of the 65 kDa species. We now know that liver *Gpx*1 transcripts are dramatically reduced in liver by Se deficiency to 10% of Se-adequate levels (35, 38, 44), providing an explanation for the blunting of ^75^Se incorporation into GPX1 in Se-deficient rats. Se repletion studies show that it takes 24 h to substantially raise liver GPX1 activity (43), further explaining the observed delay to 24 h in achieving the maximal ^75^Se incorporation into liver GPX1 (Figure 4). The failure to see increased ^75^Se incorporation into Se-deficient liver (Figures 7A,D) further shows that the administered ^75^Se was as a tracer dose which did not substantially raise total Se status. High Se status (2 μg Se/g) markedly diminished the ^75^Se labeling of both GPX1 and the 65 kDa species, illustrating additional dilution of the tracer ^75^Se (Figures 7C,F).

Our studies in this rat model show that more than half of the selenoprotein transcripts are not significantly decreased by Se deficiency; *Txnrd*1 mRNAs are only decreased to 60% of Se-adequate levels (35), This can explain why sustained ^75^Se incorporation into the 65 kDa species was observed starting at 1 h in Se-deficient rat liver. Similarly, transcripts for liver SELENOP liver are not decreased in Se deficiency, explaining the rapid labeling of plasma SELENOP by 3 h (Figure 3). *Gpx*3 transcripts in kidney are also non-significantly decreased only to 60% of Se-adequate levels (35), supporting the appearance of ^75^Se-labeled GPX3 in plasma at 24 h. Thus, the subsequent research on selenoprotein expression and regulation of selenoprotein transcripts since these tracer studies were conducted provides supporting rationale and insight into observed patterns of ^75^Se incorporation into selenoproteins.

Basic biochemical studies have shown that SeMet is readily acylated to Met-tRNA and is incorporated into proteins in place of Met (19). Nutritional studies have further shown that marginal dietary Met increases deposition of SeMet into body proteins and decreases release of Se for tissue GPX1 synthesis (17). In the present studies, feeding a marginal-Met vs. Met-adequate diet for 1 wk prior to tracer [^75^Se]SeMet injection had minimal effect of labeling of plasma SELENOP or GPX3, which indicated there was sufficient SeMet degradation to maintain the flux of Se into these species in Se-adequate rats. Similarly, high Met feeding for 1 wk also exerted at most small changes on SELENOP and GPX3 labeling. In liver in contrast, feeding a marginal Met diet for 1 wk prior to ^75^Se injection increased labeling of liver GPX1, suggesting increased catabolism of SeMet to the Se precursor used for selenoprotein synthesis; high Met feeding for 1 wk had little effect on labeling of liver GPX1, perhaps because additional Se was incorporated into plasma SELENOP. Overall, feeding these varied Met diets to older rats for just 1 week did not have as dramatic effects as was found in longer non-tracer studies in young rats, or in studies on utilization of stored SeMet in general body tissues to provide Se for GPX1 synthesis (13, 16, 17).

The hidden story in these experiments is the extent of loss of ^75^Se when tissue extracts were subjected to 2-mercaptoethanol treatment followed by SDS/PAGE. In liver and kidney at 1 and 3 h after ^75^Se injection, especially in rats fed 0.2, 2, and 5 μg Se/g, there was an increasingly small amount of the cytosolic ^75^Se detected as selenoprotein ^75^Se; in rats fed 5 μg Se/g at 1 and 3 h, <5% of liver cytosolic ^75^Se and <20% of kidney cytosolic ^75^Se was present in the selenoproteins retained in the SDS/PAGE gels. When these studies were conducted, we presumed that the missing ^75^Se was low-MW intermediates on the pathways to selenoprotein incorporation or to formation of methylated excretion products (26). With our recent finding that more Se is present as selenosugars than is present as Sec even in Se-adequate turkey liver (33), the implication is that the missing ^75^Se in these rat studies may have initially been selenosugars linked via selenodisulfide linkages nonspecifically to cysteine residues in high-MW general proteins. These species would be released by the 2-mercaptoethanol treatment and swept off at the bottom of the gel. Similarly, low MW selenosugar species such as CH_3_-SeGalNac and GS-SeGalNac would be released as well. In rats fed 2 or 5 μg Se/g vs. 0.2 μg Se/g, there was even more missing ^75^Se in rats, suggesting that increased quantities of these species are present in rat liver and kidney cytosols. The levels of these species in microsomal, mitochondrial, and nuclear fractions are completely unknown at present, as the turkey liver studies were done on extracts of frozen tissue that would have included all subcellular organelles. Lastly, the increased retention of ^75^Se in the gels at 24 and 72 h in liver and kidney, vs. 1 and 3 h, suggests that there may be rapid flux or turnover of Se within these missing, hypothetical, selenosugar pools of Se.

Low-MW selenosugars have been identified in animal tissues by multiple investigators, but they were always reported as being found in low-MW fractions. The discoverers of CH_3_-SeGalNac in urine also reported separation of liver cytosol into a high-MW protein-containing fraction and a low-MW fraction by ultrafiltration, but reported CH_3_-SeGalNac only in the low-MW fraction (32, 45). Other researchers used HPLC as the first step for plasma and tissue cytosol analysis and found late-eluting low-MW species that were identified as CH_3_-SeGalNac and GSH-SeGalNac. These researchers also found broad early-eluting HPLC peaks that were described as containing high-MW selenoproteins/Se-binding proteins, but none of these reports recognized that the high-MW protein fractions could also contain selenosugars (46–49). Takahashi and colleagues (50) used stable isotope mass spectroscopy to identify GSH-SeGalNac and CH_3_-SeGalNac in serum, liver, and kidney in Se-deficient rats given non-tracer doses of nine different selenocompounds, but they also showed uncharacterized broad high-MW Se-containing protein peaks in the HPLC profiles (50). Thus, the high-MW selenosugar-decorated proteins in turkey liver appears to be the first characterization of what might be missing in our SDS/PAGE gel profiles of rat selenoproteins.

## Conclusions

In summary, these studies show that there is very rapid Se metabolism from a diverse set of selenocompounds to the common intermediate used for synthesis and incorporation of ^75^Se into the major selenoproteins in a variety of tissues. Collectively, these studies emphasize that this wide variety of selenocompounds are not uniquely or preferentially metabolized to provide Se for selenoprotein incorporation. Furthermore, examination of the SDS/PAGE selenoprotein profiles shows that synthesis of selenoproteins is only part of the full Se metabolism story. The missing ^75^Se species, especially at early timepoints, are likely to be low-MW and high-MW selenosugars and related precursors. Differential metabolism of various selenocompounds into different selenosugar species may occur; these species may be involved in the prevention of cancer or other diseases linked to Se status and may be associated with Se toxicity. Studies similar to these presented here, and characterization of the Se species in tissues by HPLC-MS, will be needed to more fully flesh out the complete metabolism of selenium.

## Data Availability Statement

The original contributions presented in the study are included in the article/supplementary material, further inquiries can be directed to the corresponding authors.

## Ethics Statement

The animal study was reviewed and approved by Animal Care and Use Committee, University of Arizona (A3248 #86-0172 and #86-0357) Animal Care and Use Committee, University of Missouri (A3394 #1425).

## Author Contributions

All authors listed have made a substantial, direct and intellectual contribution to the work, and approved it for publication.

## Conflict of Interest

The authors declare that the research was conducted in the absence of any commercial or financial relationships that could be construed as a potential conflict of interest.
